# Multihost Transmission of *Schistosoma mansoni* in Senegal, 2015–2018

**DOI:** 10.3201/eid2606.200107

**Published:** 2020-06

**Authors:** Stefano Catalano, Elsa Léger, Cheikh B. Fall, Anna Borlase, Samba D. Diop, Duncan Berger, Bonnie L. Webster, Babacar Faye, Nicolas D. Diouf, David Rollinson, Mariama Sène, Khalilou Bâ, Joanne P. Webster

**Affiliations:** Royal Veterinary College, University of London, Hatfield, UK (S. Catalano, E. Léger, A. Borlase, J.P. Webster);; Université Cheikh Anta Diop, Dakar, Senegal (C.B. Fall, B. Faye);; Big Data Institute, University of Oxford, Oxford, UK (A. Borlase);; Université Alioune Diop de Bambey, Bambey, Senegal (S.D. Diop);; Wellcome Sanger Institute, Hinxton, UK (D. Berger);; Natural History Museum, London, UK (B.L. Webster, D. Rollinson);; Université Gaston Berger, Saint-Louis, Senegal (N.D. Diouf, M. Sène);; Institut de Recherche pour le Développement, Dakar (K. Bâ)

**Keywords:** *Schistosoma*, *Schistosoma mansoni*, zoonoses, parasites, *Rodentia*, rodents, *Mastomys huberti*, snail, *Biomphalaria pfeifferi*, children, West Africa, Senegal, Senegal River Basin, Lac de Guiers, infectious diseases, One Health, molecular epidemiology, reservoir, transmission, definitive host, evolution, multihost system, schistosomiasis

## Abstract

In West Africa, *Schistosoma* spp. are capable of infecting multiple definitive hosts, a lifecycle feature that may complicate schistosomiasis control. We characterized the evolutionary relationships among multiple *Schistosoma mansoni* isolates collected from snails (intermediate hosts), humans (definitive hosts), and rodents (definitive hosts) in Senegal. On a local scale, diagnosis of *S. mansoni* infection ranged 3.8%–44.8% in school-aged children, 1.7%–52.6% in *Mastomys huberti* mice, and 1.8%–7.1% in *Biomphalaria pfeifferi* snails. Our phylogenetic framework confirmed the presence of multiple *S. mansoni* lineages that could infect both humans and rodents; divergence times of these lineages varied (0.13–0.02 million years ago). We propose that extensive movement of persons across West Africa might have contributed to the establishment of these various multihost *S. mansoni* clades. High *S. mansoni* prevalence in rodents at transmission sites frequented by humans further highlights the implications that alternative hosts could have on future public health interventions.

The collective image of schistosomiasis in Africa remains that of a mainly human-driven disease; schistosomiasis inflicted a burden of >2.5 million disability-adjusted life-years in 2016 and required that ≈200 million persons be treated with preventive chemotherapy in 2017 (*1*). As pledged by the World Health Organization (*2*), the goal to eliminate schistosomiasis as a public health problem by 2030 can only be achieved through transdisciplinary programs that improve sanitation and hygiene and provide access to safe water sources, health education, and chemotherapeutic treatments for at-risk populations. Furthermore, answers on the host specificity of human schistosomes and the impact of multihost transmission on disease control strategies remain imperative (*3*). In Asia, vertebrate reservoirs for *Schistosoma japonicum* (largely ruminants, rodents, and other mammals) play a crucial role in perpetuating the transmission of this zoonotic parasite, even under strong multisectoral control pressures (*4*,*5*). Likewise, in the Caribbean and South America, where evidence supports the introduction of *Schistosoma mansoni* from West Africa via the transatlantic slave trade (*6*), rodent populations have become the main reservoirs of *S. mansoni*; transmission in this region can be maintained in absence of human activity (*7*,*8*).

The magnitude of *Schistosoma* zoonotic transmission, in which both domestic animals and wildlife are active participants, is yet to be determined in endemic countries across Africa. Sporadic investigations have attempted to answer whether schistosomes infecting humans are zoonotic and which, if any, other vertebrate species might be acting as definitive hosts (*9*–*11*). The emergence (or discovery) of hybridization events involving *S. mansoni*, *Schistosoma haematobium*, and other *Schistosoma* spp. in livestock and wildlife has raised the profile of these definitive hosts and the schistosomes they harbor (*12*,*13*). The interspecific interactions between *Schistosoma* spp. and the potential involvement of domestic and wild vertebrates in the transmission dynamics of these species might partially be a consequence of anthropogenic changes, loss of ecologic barriers, and movement of communities between endemic areas (*12*).

In 1986, the Diama Dam became operational and transformed the Senegal River Basin. The rice and sugarcane industries benefitted extensively from this change in land use, and the guaranteed freshwater supply favored the expansion of subsistence farming and livestock husbandry. In addition, communities attracted by employment opportunities migrated to the region, in particular to the town of Richard Toll and villages nearby the lake Lac de Guiers in northern Senegal (*14*,*15*). However, these anthropogenic changes in the area rapidly led to the first outbreaks of schistosomiasis in the early 1990s (*16*). As of April 2020, both intestinal schistosomiasis (caused by *S. mansoni*) and urogenital schistosomiasis (caused by *S. haematobium* and schistosome hybrids) remain endemic, with co-infections commonly observed across the Senegal River Basin (*17*). Records show a prevalence of 32%–40% for *S. mansoni* and 77%–81% for *S. haematobium* and schistosome hybrids in school-aged children and adults inhabiting towns surrounding Lac de Guiers and along the Senegal River (*18*,*19*). In this scenario, the role of animal hosts in the epidemiology of schistosomiasis is unclear. Wild rodents and humans seem to share the same *Schistosoma* species and hybrids at transmission foci (*20*,*21*). However, whether these schistosomes are truly multihost parasites or, in contrast, they have followed diverging evolutionary pathways indicative of definitive host specialization remains to be determined. Focusing on the regions of Richard Toll and Lac de Guiers, our objectives were to examine the evolutionary relationships and host use among *Schistosoma* isolates and the potential for rodent-to-human spillover.

## Materials and Methods

### Small Mammal Trapping

During October–December 2017, we captured small mammals at 21 sites that represented *Schistosoma* spp. transmission foci frequented by humans and their livestock because they are access points to fresh water (Appendix Figure 1). These study sites were situated within or adjacent to villages on the shores of Lac de Guiers and were considered independent from each other for trapping purposes; the shortest distance between adjacent sites was ≈500 m, greater than the maximum home range of endemic species (*22*). We baited locally made wire-mesh live traps (26 × 10 × 10 cm) with peanut butter and placed them in lines of 14–22 traps at intervals of 5 m adjacent to bodies of water in riparian habitats where reeds (*Phragmites* sp. and *Typha* sp.) were the dominant vegetation. We set traps each evening before dusk and inspected them the following morning after dawn for 2 consecutive nights per study site. We calculated the relative abundance of trapped species (no. animals captured/no. active traps) per night for each trap site (*23*). 

We euthanized small trapped mammals with an intraperitoneal injection of sodium thiopental (300 mg/kg body weight) and confirmed their deaths by cervical dislocation and the absence of pedal reflex. We recorded each animal’s species (based on morphologic identification), sex, age class, and anatomic measurements at postmortem examination (Appendix); dissected their thoracic and abdominal organs separately; and visually inspected these organs for helminths. We separated *Schistosoma* pairs, preserved them in separate vials containing 95% ethanol, and stored them at –20°C. We macerated dissected livers and large intestines of *Schistosoma*-positive hosts through 300 µm metal sieves using bottled spring water to hatch miracidia and then collected the free-swimming miracidia onto Whatman Indicating FTA Classic Cards (GE Healthcare Life Sciences, https://www.gelifesciences.com) for DNA storage and molecular analysis (*24*,*25*). We archived *Schistosoma* miracidia and adult worms in the Schistosomiasis Collection at the Natural History Museum (*26*). 

### Human and Snail Surveys

During October 2017–January 2018, as part of a large-scale program on the transmission dynamics of *Schistosoma* spp. across Senegal, we conducted a survey for parasites among randomly selected school-aged children (5–17 years of age, n = 290) and self-selected adults (18–78 years of age, n = 40) in the region of Richard Toll and Lac de Guiers. Each person provided 1 fecal sample; we diagnosed *Schistosoma* infections when eggs were observed in duplicate Kato-Katz thick smears (*27*). We processed each *Schistosoma*-positive fecal sample (30 g or the whole sample if <30 g) separately using the miracidial hatching technique (*25*) and pipetted the free-swimming miracidia onto Whatman Indicating FTA Classic Cards for DNA storage and molecular analysis (*24*). We archived *Schistosoma* miracidia in the Schistosomiasis Collection at the Natural History Museum (*26*).

During November 2015–April 2018, we sampled open freshwater sources within and nearby villages where we conducted surveys with human volunteers to identify snails acting as intermediate hosts of *Schistosoma* parasites. Throughout 5 surveys, we applied standardized protocols in malacology, determined species of collected snails, and identified cercarial shedding to diagnose infections (*28*). We pipetted free-swimming *Schistosoma* cercariae, which we identified using a morphologic key (*29*), onto Whatman Indicating FTA Classic Cards for DNA storage and molecular analysis (*24*). We archived *Schistosoma* cercariae in the Schistosomiasis Collection at the Natural History Museum (*26*).

### Molecular Analyses

We extracted DNA of individual adult schistosomes using the DNeasy Blood and Tissue Kit (QIAGEN, https://www.qiagen.com) following the manufacturer’s instructions and extracted the DNA of miracidia and cercariae stored on Whatman Indicating FTA Classic Cards as previously described (*30*). We analyzed the following genomic regions because they are highly informative for phylogenetic identification and classification (*31*): the internal transcribed spacers (ITS) of the nuclear rDNA, the mitochondrial 12S rRNA gene, cytochrome *c* oxidase subunit 1 (*cox1*) and subunit 3 (*cox3*) genes of the mitochondrial DNA (mtDNA), and NADH dehydrogenase subunit 4 (*nad4*) and subunit 3 (*nad3*) genes of the mtDNA. We amplified these regions using 25-µL reactions containing 2.5 µL of 10× buffer, 200 µM of dNTPs, 0.5 µM of each primer, 0.2 units of KOD XL DNA Polymerase (EMD Millipore Corporation, https://www.emdmillipore.com), and 2 µL of DNA template (Appendix Tables 1 and 2). We purified and sequenced PCR products using Eurofins Genomics (https://www.eurofinsgenomics.com) and then edited and assembled contigs using CodonCode Aligner 8.0.1 (https://www.codoncode.com/index.htm). We aligned the noncoding ITS and 12S regions using MAFFT v7 (*32*) with automated selection of parameters and aligned the protein-coding mtDNA genes (i.e., *cox1*, *cox3*, *nad4*, and *nad3*) with respect to their amino acid translations using MACSE (*33*) as implemented in CodonCode Aligner 8.0.1. Molecular sequences from the *S. mansoni* samples are deposited in GenBank (accession nos. MN593375–434).

### Phylogenetic Approach

We concatenated the 12S rRNA gene and the 4 protein-coding mtDNA genes of each *S. mansoni* specimen (i.e., adult worms and miracidia from rodents, miracidia from humans, and cercariae from snails), as well as those from *S. mansoni* specimens previously collected from Hubert’s multimammate mice (*Mastomys huberti*) and Nile grass rats (*Arvicanthis niloticus*) in Senegal (*21*). In addition, we also obtained and concatenated the respective sequences from publicly available genomes of *S. mansoni* previously isolated from school-aged children in Uganda (*34*) and *Schistosoma rodhaini* from an undetermined intermediate host in Burundi (*6*). In brief, we downloaded an *S. mansoni* reference genome (GenBank accession no. SAMEA2272516) from WormBase ParaSite (*35*) and aligned the 5 specified mitochondrial genes with those of *S. mansoni* from Uganda and *S. rodhaini* from Burundi using BWA-MEM version 0.7.17 (Li H, unpub. data, https://arxiv.org/abs/1303.3997v2). For each sample, we used the Genome Analysis Toolkit (*36*) tools HaplotypeCaller version 3.6.0 to perform variant calling and FastaAlternateReferenceMaker version 3.6.1.0 to replace reference bases with single-nucleotide polymorphisms at variation sites.

We implemented maximum-likelihood analyses in RAxML version 8.2 (*37*) and Bayesian inference analyses in MrBayes 3.2.6 (*38*). Across 4 partitions (noncoding positions and protein-encoding first, second, and third codon positions), we selected the generalized time-reversible substitution model with rate heterogeneity for both maximum-likelihood and Bayesian inference. Bootstrap resampling was automatically arrested within the maximum-likelihood analysis. We performed Bayesian inference analysis using 2 independent Markov chain Monte Carlo runs including 4 chains and 10 million generations, sampling every 10,000 generations, and discarding the first 25% of trees as burn-in (Appendix).

We analyzed the temporal structure of the data by using Bayesian inference analysis and specifying independent Hasegawa-Kishino-Yano substitution models with rate heterogeneity across the 4 partitions, a coalescent constant population tree prior with default settings, and a strict clock model in BEAST 2.5.1 (*39*). We based divergence dating on previous estimates of mutation rates (8.1 × 10^–9^ substitutions/site/year) per generation time (0.2 years) that were determined by using whole-genome *S. mansoni* sequences (*6*). We inferred the resulting uniform clock rate prior of 4.05 × 10^–8^ substitutions/site/year. We computed 2 independent Markov chain Monte Carlo runs including 10 million generations, sampling every 1,000 generations, and discarding the first 10% of trees as burn-in. We inspected convergence and effective sample size values >200 using Tracer version 1.7.1 (https://beast.community/tracer) and generated the maximum clade credibility tree using TreeAnnotator version 2.5.1 (https://beast.community/treeannotator). We tested the association between phylogenetic clustering and geographic structure of *S. mansoni* isolates in BaTS (*40*) by implementing 1,000 null replicates, 5 discrete states, and an initial burn-in period of 10% (Appendix).

### Ethical Considerations

We obtained informed written consent from all human participants or their legal guardians. All infected persons were treated with praziquantel 40 mg/kg either at school or at home. After explicit consent from local authorities and land owners, we targeted our small mammal trapping activities on animal populations classified as least concern by the International Union for the Conservation of Nature Red List. We recorded the trapping of nontarget animals (i.e., unidentified birds and anuran amphibians) and immediately released them at their point of capture. The examined animals were treated in accordance with published guidelines on animal welfare and the use of wildlife in research (*41*). All investigations were approved by the Comité National d’Ethique pour la Recherche en Santé of Senegal (reference no. SEN15/68), the Imperial College Research Ethics Committee of the United Kingdom (reference no. 03.36), and the Clinical Research Ethical Review Board of the Royal Veterinary College of the United Kingdom (reference nos. 2015-1327 and 2016-1505).

## Results

A total of 1,618 traps were set over the course of 27 consecutive nights, and 195 *M. huberti* mice, 42 *A. niloticus* rats, and 14 *Crocidura* shrews were trapped and examined (Appendix Figure 2). We detected *Schistosoma* trematodes in 16 (8.2%) *M. huberti* mice (Appendix Table 3), specifically in the mesenteric vessels (in 81.2% of infected mice) and the portal system (in 68.7% of infected mice). On a local scale, 1.7%–52.6% of *M. huberti* mice were infected with *S. mansoni* (Table 1). In contrast, we did not observe *Schistosoma* infections in *A. niloticus* rats and *Crocidura* shews or at the dissection of the urogenital systems of any animals trapped during this study (Appendix Table 3). Miracidial hatching was successful for 8 of 16 infected *M. huberti* mice. No association was found between *Schistosoma* infection prevalence and *M. huberti* mice sex or age (Appendix). All adult schistosomes and miracidia from infected rodents were identified as *S. mansoni* on the basis of molecular analyses.

**Table 1:** *Schistosoma mansoni* infection rate and intensity by host and study site, Senegal, 2015–2018*

Study site	*Mastomys huberti* mice			*Arvicanthis niloticus* rats			School-aged children		*Biomphalaria pfeifferi*, snails, no. infected/ total no. (%)
	No. infected/ total no. (%)	Median (range) infection intensity		No. infected/ total no. (%)	Median (range) infection intensity		No. infected/ total no. (%)	Median (range) infection intensity	
Didjiery†	0/12	NA		0/69	NA		6/17 (35.3)	180 (12–408)	0/111
Ganket	2/4 (50.0)	18.5 (5–32)		0/4	NA		NA	NA	NA
Gueo	10/19 (52.6)	14 (2–64)		NA	NA		NA	NA	NA
Keur Momar Sarr	1/19 (5.3)	2		NA	NA		NA	NA	NA
Mbane†	0/60	NA		0/34	NA		1/26 (3.8)	264	1/55 (1.8)
Merina Guewel	1/12 (8.3)	2		NA	NA		6/16 (37.5)	42 (12–108)	NA
Nder†	1/60 (1.7)	2		0/11	NA		5/44 (11.4)	12 (12–24)	6/84 (7.1)
Ndombo	NA	NA		NA	NA		5/101 (5.0)	12 (12–24)	0/5
Richard Toll†	0/10	NA		1/73 (1.4)	4		13/29 (44.8)	180 (24–1,656)	0/4
Temeye†	8/43 (18.6)	4 (2–35)		0/4	NA		1/21 (8.3)	12	2/75 (2.7)
Thiago†	0/4	NA		NA	NA		NA	NA	0/2

*Only study sites where infected hosts were detected are included. Infection intensities were calculated by using eggs per gram of fecal samples for school-aged children and number of adult worms in rodents (*M. huberti* and *A. niloticus*). Infection intensity was not quantified for *B. pfeifferi* snails (intermediate host). NA, not applicable. †For rodents, values include data previously reported (*21*).

A total of 290 school-aged children were examined by duplicate Kato-Katz thick smears, and 37 (12.8%) had *S. mansoni* infections. We performed miracidial hatching on a randomly selected subset of *Schistosoma*–positive fecal samples and collected miracidia from samples from 4 infected persons. Molecular analysis confirmed the identification of *S. mansoni*. In contrast, none of the 40 adults examined by duplicate Kato-Katz thick smears had *S. mansoni* infections. On a local scale, 3.8%–44.8% of school-aged children were infected with *S. mansoni* (Table 1). A total of 407 *Biomphalaria pfeifferi* snails were observed for cercarial shedding and 9 (2.2%) had *S. mansoni* infections, which were identified by using molecular tools. On a local scale, 1.8%–7.1% of *B. pfeifferi* snails were infected with *S. mansoni* (Table 1).

The dataset including ITS, 12S rRNA, and the protein-coding mtDNA sequences (i.e., *cox1*, *cox3*, *nad4*, and *nad3*) of *S. mansoni* from school-aged children, rodents, and *B. pfeifferi* snails (Table 2) showed no intraspecific variability within the ITS alignment (914 bp), whereas variability was present within the 12S (760 bp, polymorphism <0.52%) and concatenated mtDNA (2,874 bp, polymorphism <0.77%) gene alignments. Intraspecific single-nucleotide polymorphisms within protein-coding mtDNA genes represented nonsynonymous amino acid substitutions in 4.4% (35/796) of codons, and saturation at model-corrected genetic distances was not detected (Appendix Figure 3). Maximum-likelihood and Bayesian inference analyses of the concatenated 12S and mtDNA gene sequences yielded consensus trees with congruent topologies, including different multihost *S. mansoni* lineages (Appendix Figure 4). The presence of multiple, well-supported *S. mansoni* clades within Senegal, 4 of which included samples collected from both humans and rodents, was confirmed by a phylogenetic analysis constructed by using a strict molecular clock (Figure, panel A; Appendix Figure 5). Different *S. mansoni* lineages prevalent in the Senegal River Basin diverged between 0.13 (95% highest posterior density interval [HPDI] 0.11–0.16) and 0.02 (95% HPDI 0.01–0.03) million years ago (MYA). Using uniform clock rate prior (4.05 × 10^–8^ substitutions/site/year), we determined that divergence between the sampled *S. mansoni* parasites from Uganda and Senegal occurred ≈0.19 (95% HPDI 0.15–0.23) MYA, whereas the speciation of *S. rodhaini* may have occurred ≈1.14 (95% HPDI 0.95–1.35) MYA. The association index, parsimony score, and monophyletic clade metrics of *S. mansoni* within Senegal were not significant in BaTS (p>0.05). These findings strongly support the null hypothesis of random phylogenetic trait associations and, therefore, that *S. mansoni* clades are not associated with the geographic structure on a local scale (Figure, panel B). 

**Table 2:** *Schistosoma* specimens from Senegal, Uganda, and Burundi, 2002–2018, included in phylogenetic analysis to determine if certain *S. mansoni* clades use multiple definitive hosts*

Source	Parasite	Stage†	No. isolates	Sampling locality	Isolation year	GenBank or ENA accession no.
Reference	*S. rodhaini*	Adult	1	Burundi	2002	SAMEA1979799
Definitive host						
Human	*S. mansoni*	Miracidium	4	Mayuge, Uganda	2014	SAMEA5366708, SAMEA5366733, SAMEA5366938, SAMEA5367037
	*S. mansoni*	Miracidium	1	Tororo, Uganda	2014	SAMEA5366700
	*S. mansoni*	Miracidium	1	Nder, Senegal	2017	MN593383–6
	*S. mansoni*	Miracidium	3	Temeye, Senegal	2017	MN593387–90
	*S. mansoni*	Miracidium	3	Didjiery, Senegal	2018	MN593375–82
*Mastomys huberti* mouse	*S. mansoni*	Adult	1	Nder, Senegal	2016	NA
	*S. mansoni*	Adult	10	Gueo, Senegal	2017	MN593427–34
	*S. mansoni*	Miracidium	1	Gueo, Senegal	2017	NA
	*S. mansoni*	Adult	2	Ganket, Senegal	2017	MN593419–22
	*S. mansoni*	Miracidium	2	Ganket, Senegal	2017	MN593411–4
	*S. mansoni*	Adult	2	Temeye, Senegal	2017	NA
	*S. mansoni*	Miracidium	4	Temeye, Senegal	2017	MN593415–8
	*S. mansoni*	Adult	2	Merina Guewel, Senegal	2017	MN593423–6
	*S. mansoni*	Miracidium	2	Merina Guewel, Senegal	2017	NA
*Arvicanthis niloticus* rat	*S. mansoni*	Adult	3	Richard Toll, Senegal	2016	MN593407–10
Intermediate host						
*Biomphalaria pfeifferi* snail	*S. mansoni*	Cercaria	1	Mbane, Senegal	2015	MN593391–4
	*S. mansoni*	Cercaria	4	Temeye, Senegal	2016	MN593395–402
	*S. mansoni*	Cercaria	2	Nder, Senegal	2016	MN593403–6

*ENA, European Nucleotide Archive; NA, not applicable.

**Figure:**
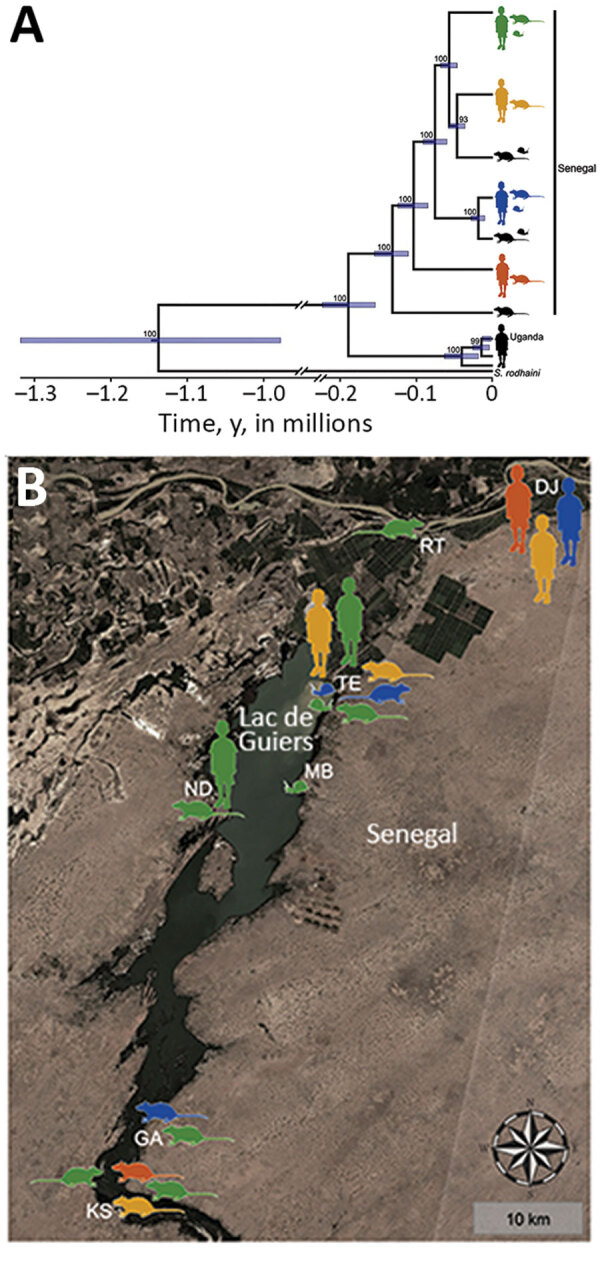
Phylogenetic analysis and geographic locations of *Schistosoma mansoni* lineages isolated from both humans and rodents (colored silhouettes) or from a single definitive host (black silhouettes), Senegal. Rodent silhouettes represent *Mastomys huberti* mice or *Arvicanthis niloticus* rats and snail silhouettes represent *Biomphalaria pfeifferi* snails (intermediate host). A) Bayesian tree made by using a strict molecular clock and the concatenated mitochondrial 12S rRNA and 4 protein-coding mitochondrial DNA gene sequences. *Schistosoma rodhaini* and *S. mansoni* samples from school-aged children in Uganda were included in the analysis. Posterior probabilities and 95% highest posterior density intervals (blue rectangles) are indicated for each node. Branches with nodal support <90% were collapsed. For complete tree, see Appendix Figure 5. B) Geographic locations of multihost *S. mansoni* lineages, Richard Toll and Lac de Guiers regions. Satellite imagery from Sentinel Hub (Sinergise, https://www.sentinel-hub.com) was used as the base layer. DJ, Didjiery; GA, Ganket; KS, Keur Momar Sarr; MB, Mbane; ND, Nder; RT, Richard Toll; TE, Temeye.

## Discussion

In this study, we provide direct evidence of the zoonotic nature of *S. mansoni* in West Africa, revealing a potential ecologic cause for human reinfection after chemotherapeutic treatment. Our phylogenetic approach demonstrated that *S. mansoni* lineages responsible for intestinal schistosomiasis in humans also exploit rodent populations as reservoirs at transmission sites frequented by humans; prevalence could be as high as 52.6% in *M. huberti* mice at these sites. Therefore, we exclude the presence of an independent sylvatic life cycle and host specialization for *S. mansoni* in the Senegal River Basin. The phylogenetic similarity between parasite isolates collected from humans, rodents, and freshwater snails indicates that host use has not played a prominent role in the evolutionary pathway of *S. mansoni* in this region. Similar results were obtained during the analysis of specimens from different regions and hosts within the geographic distribution of *S. mansoni*, suggesting that murine isolates did not constitute monophyletic assemblages (*42*).

This lack of a geographic structure for *S. mansoni* on the local scale might be caused by disease foci of recent origin or the rapid dissemination of *S. mansoni* across the Senegal River Basin, which probably occurred as a result of the land-use changes associated with the Diama Dam construction and transport infrastructure development (*14*–*16*). Furthermore, 3 decades of endemicity and the extensive movement of communities from within Senegal and other countries of West Africa could have substantially contributed to *S. mansoni* lineage diversification and gene flow in the Lac de Guiers region (*14*,*43*). The different *S. mansoni* clades detected herein might have diverged between 0.13 + 0.03 MYA and 0.02 + 0.01 MYA, firmly corroborating the hypothesis of their ramification from a common precursor during ancestral times. Multiple introduction events of various parasite populations could indicate that *M. huberti* mice and other rodent populations inhabiting periaquatic ecosystems act as competent alternative hosts for *S. mansoni* in many endemic areas across sub-Saharan Africa. The mainly nocturnal activity of *M. huberti* mice (vs. diurnal activity of *A. niloticus* rats) (*22*) may support the presence of different *S. mansoni* chronotypes characterized by differing circadian rhythms of cercarial emergence (*44*). Therefore, the risk for infection among local communities might not be limited to just the warmest hours of the day (diurnal transmission) but also extend to the early morning and late afternoon (crepuscular transmission). The high excretion rates of *S. mansoni* eggs by *M. huberti* mice during experimental infections (median intensity 720 eggs/g fecal sample) (*45*) and field observations (median intensity 262 eggs/g fecal sample) (*46*) are a warning about the potential contamination of freshwater bodies by parasitized rodents.

In our study, fully resolved spatial and temporal dynamics could not be determined. Future incorporation of *S. mansoni* sequences from multiple endemic regions across West Africa and Africa as a whole might help decipher the origin and radiation pattern of the various lineages observed in the Richard Toll and Lac de Guiers areas. Furthermore, the temporal estimates of *S. mansoni* evolution displayed herein should be interpreted with caution. The molecular clock calibration relied on previous estimates of the mutation rate and generation time calculated by using whole-genome *S. mansoni* data across its known geographic distribution (*6*). However, our reconstruction of the divergence between *S. rodhaini* and *S. mansoni* (1.14 + 0.20 MYA) differs from previous dating (0.13 + 0.02 MYA and 2.80 + 0.19 MYA) (*6*,*42*). This conundrum highlights that further evidence is needed to characterize the evolutionary history within the genus *Schistosoma*. The application of a single calibration method in divergence dating remains subject to time-dependent bias if not integrated by ancestral DNA, fossil records, or biogeographic events (*47*).

The zoonotic *S. japonicum* in Asia illustrates the pivotal role that animal reservoirs and multihost dynamics have as drivers of pathogen transmission and human reinfection, even after decades of multifaceted interventions (*4*,*5*). With the presence of multiple multihost *S. mansoni* lineages characterized by different divergence times circulating across the Senegal River Basin, our results support a similar scenario for *S. mansoni* in sub-Saharan Africa. Therefore, the parasite should be acknowledged as zoonotic, and public health campaigns must be planned considering the availability of alternative hosts (including wildlife, although *S. mansoni* prevalence in wildlife reservoirs can markedly vary) when transmission is maintained despite repeated interventions. The implementation of coprologic and DNA-based diagnostics within nonlethal sampling schemes can directly facilitate targeted surveillance where rodents might be contributing to the transmission of *S. mansoni*, other *Schistosoma* spp., and hybrids. However, the results of our study and previous surveys in endemic settings of Senegal (*21*) and Corsica, France (*48*), support the role of rodents as accidental (rather than maintenance) hosts of the *Schistosoma* hybrids responsible for urogenital schistosomiasis. Furthermore, although evidence suggests that rodents could be competent hosts of *Schistosoma bovis* (typically a schistosome of ruminants) across sub-Saharan Africa (*11*,*21*), we did not isolate any during this survey.

In conclusion, the multihost transmission dynamics of *S. mansoni* promote the recruitment of various definitive hosts spatially and temporally overlapping at transmission sites in the region of Lac de Guiers. In sub-Saharan Africa, the role of nonhuman vertebrates in the epidemiology of *Schistosoma* species and hybrids has yet to be fully determined, considering these could be spillover hosts incapable of maintaining transmission by themselves. However, our study supports that rodents have the potential to act as true reservoirs of *S. mansoni* and influence the evolution of this parasite (i.e., by providing opportunities for host switching and genetic exchange), which could thwart attempts to control or interrupt transmission of *S. mansoni* in human populations (*3*,*12*). Nevertheless, the presence of zoonotic pathogens in their animal reservoirs should not be considered synonymous with human disease risk, but rather a measure of underlying transmission potential, which is itself mediated by many additional intersecting ecologic and social drivers (*19*,*49*). The extent to which rodents contribute to the zoonotic transmission of *S. mansoni* and *Schistosoma* hybrids remains a question to be further developed by epidemiologic surveys, mathematical modelling, and genomics. As we move our efforts from disease control toward interruption of *S. mansoni* transmission and local elimination, the implication of alternative hosts in disease dynamics will be crucial and threaten to undermine future chemotherapeutic-focused interventions on local scales. Cross-disciplinary initiatives between the natural resource and public health sectors, including the long-term establishment of regional expertise, can be used to guide preventive measures not only for schistosomiasis but also for other rodentborne zoonoses across Africa and beyond. 

Appendix More information on multihost transmission of *Schistosoma mansoni* in Senegal, 2015–2018.

## References

[1] World Health Organization. Schistosomiasis and soil-transmitted helminthiases: numbers of people treated in 2017. Wkly Epidemiol Rec. 2018;93:681–92. https://www.who.int/publications-detail/who-wer9350

[2] World Health Organization. Ending the neglect to attain the sustainable development goals: a road map for neglected tropical diseases 2021–2030. 2020 Feb [cited 2020 Feb 24]. https://www.who.int/neglected_diseases/Ending-the-neglect-to-attain-the-SDGs--NTD-Roadmap.pdf?ua=1

[3] Colley DG, Loker ES. New tools for old questions: how strictly human are “human schistosomes”—and does it matter? J Infect Dis. 2018;218:344–6. 10.1093/infdis/jiy030 29365121

[4] Rudge JW, Webster JP, Lu DB, Wang TP, Fang GR, Basáñez MG. Identifying host species driving transmission of schistosomiasis japonica, a multihost parasite system, in China. Proc Natl Acad Sci U S A. 2013;110:11457–62. 10.1073/pnas.1221509110 23798418 PMC3710859

[5] Gordon CA, Kurscheid J, Williams GM, Clements ACA, Li Y, Zhou XN, et al. Asian schistosomiasis: current status and prospects for control leading to elimination. Trop Med Infect Dis. 2019;4:40. 10.3390/tropicalmed4010040 30813615 PMC6473711

[6] Crellen T, Allan F, David S, Durrant C, Huckvale T, Holroyd N, et al. Whole genome resequencing of the human parasite *Schistosoma mansoni* reveals population history and effects of selection. Sci Rep. 2016;6:20954. 10.1038/srep20954 26879532 PMC4754680

[7] Théron A, Sire C, Rognon A, Prugnolle F, Durand P. Molecular ecology of *Schistosoma mansoni* transmission inferred from the genetic composition of larval and adult infrapopulations within intermediate and definitive hosts. Parasitology. 2004;129:571–85. 10.1017/S0031182004005943 15552402

[8] Gentile R, Barreto MG, Gonçalves MM, Soares MS, D’Andrea PS. The role of wild rodents in the transmission of *Schistosoma mansoni* in Brazil. In: Rokni MB, editor. Schistosomiasis. London: IntechOpen Limited; 2012. p. 231–54. https://www.intechopen.com/books/schistosomiasis/the-role-of-wild-rodents-in-the-transmission-of-schistosoma-mansoni-in-brazil

[9] Standley CJ, Dobson AP, Stothard JR. Out of animals and back again: schistosomiasis as a zoonosis in Africa. In: Rokni MB, editor. Schistosomiasis. London: IntechOpen Limited; 2012. p. 209–30. https://www.intechopen.com/books/schistosomiasis/out-of-animals-and-back-again-schistosomiasis-as-a-zoonosis-in-africa

[10] Webster BL, Diaw OT, Seye MM, Webster JP, Rollinson D. Introgressive hybridization of *Schistosoma haematobium* group species in Senegal: species barrier break down between ruminant and human schistosomes. PLoS Negl Trop Dis. 2013;7:e2110. 10.1371/journal.pntd.0002110 23593513 PMC3617179

[11] Hanelt B, Mwangi IN, Kinuthia JM, Maina GM, Agola LE, Mutuku MW, et al. Schistosomes of small mammals from the Lake Victoria Basin, Kenya: new species, familiar species, and implications for schistosomiasis control. Parasitology. 2010;137:1109–18. 10.1017/S0031182010000041 20380765

[12] Webster JP, Gower CM, Knowles SC, Molyneux DH, Fenton A. One health - an ecological and evolutionary framework for tackling Neglected Zoonotic Diseases. Evol Appl. 2016;9:313–33. 10.1111/eva.12341 26834828 PMC4721077

[13] Léger E, Webster JP. Hybridizations within the Genus Schistosoma: implications for evolution, epidemiology and control. Parasitology. 2017;144:65–80. 10.1017/S0031182016001190 27572906

[14] Van den Broeck F, Maes GE, Larmuseau MH, Rollinson D, Sy I, Faye D, et al. Reconstructing colonization dynamics of the human parasite *Schistosoma mansoni* following anthropogenic environmental changes in northwest Senegal. PLoS Negl Trop Dis. 2015;9:e0003998. 10.1371/journal.pntd.0003998 26275049 PMC4537236

[15] Jones I, Lund A, Riveau G, Jouanard N, Ndione RA, Sokolow SH, et al. Ecological control of schistosomiasis in Sub-Saharan Africa: restoration of predator-prey dynamics to reduce transmission. In: Roche B, Broutin H, Simard F, editors. Ecology and evolution of infectious disease: pathogen control and public health management in low-income countries. Oxford: Oxford University Press; 2018. p. 236–51.

[16] Uhlir PF. Scientific data for decision making toward sustainable development: Senegal River Basin case study. Washington: The National Academies Press; 2002.

[17] Knowles SCL, Webster BL, Garba A, Sacko M, Diaw OT, Fenwick A, et al. Epidemiological interactions between urogenital and intestinal human schistosomiasis in the context of praziquantel treatment across three West African countries. PLoS Negl Trop Dis. 2015;9:e0004019. 10.1371/journal.pntd.0004019 26469347 PMC4607489

[18] Boon NAM, Van Den Broeck F, Faye D, Volckaert FAM, Mboup S, Polman K, et al. Barcoding hybrids: heterogeneous distribution of *Schistosoma haematobium* × *Schistosoma bovis* hybrids across the Senegal River Basin. Parasitology. 2018;145:634–45. 10.1017/S0031182018000525 29667570

[19] Lund AJ, Sam MM, Sy AB, Sow OW, Ali S, Sokolow SH, et al. Unavoidable risks: local perspectives on water contact behavior and implications for schistosomiasis control in an agricultural region of northern Senegal. Am J Trop Med Hyg. 2019;101:837–47. 10.4269/ajtmh.19-0099 31452497 PMC6779182

[20] Duplantier JM, Sène M. Rodents as reservoir hosts in the transmission of *Schistosoma mansoni* in Richard-Toll, Senegal, West Africa. J Helminthol. 2000;74:129–35. 10.1017/S0022149X00000172 10881283

[21] Catalano S, Sène M, Diouf ND, Fall CB, Borlase A, Léger E, et al. Rodents as natural hosts of zoonotic *Schistosoma* species and hybrids: an epidemiological and evolutionary perspective from West Africa. J Infect Dis. 2018;218:429–33. 10.1093/infdis/jiy029 29365139

[22] Granjon L, Duplantier JM. Les rongeurs de l'Afrique Sahélo-Soudanienne. Marseille (France): Muséum National d’Histoire Naturelle; 2009.

[23] Whisson DA, Engeman RM, Collins K. Developing relative abundance techniques (RATs) for monitoring rodent populations. Wildl Res. 2005;32:239–44. 10.1071/WR03128

[24] Gower CM, Shrivastava J, Lamberton PHL, Rollinson D, Webster BL, Emery A, et al. Development and application of an ethically and epidemiologically advantageous assay for the multi-locus microsatellite analysis of *Schistosoma mansoni.* Parasitology. 2007;134:523–36. 10.1017/S0031182006001685 17096873 PMC2613677

[25] Yu JM, de Vlas SJ, Jiang QW, Gryseels B. Comparison of the Kato-Katz technique, hatching test and indirect hemagglutination assay (IHA) for the diagnosis of *Schistosoma japonicum* infection in China. Parasitol Int. 2007;56:45–9. 10.1016/j.parint.2006.11.002 17188018

[26] Emery AM, Allan FE, Rabone ME, Rollinson D. Schistosomiasis collection at NHM (SCAN). Parasit Vectors. 2012;5:185. 10.1186/1756-3305-5-185 22943137 PMC3453491

[27] Katz N, Chaves A, Pellegrino J. A simple device for quantitative stool thick-smear technique in *Schistosomiasis mansoni.* Rev Inst Med Trop Sao Paulo. 1972;14:397–400. 4675644

[28] Allan F, Dunn AM, Emery AM, Stothard JR, Johnston DA, Kane RA, et al. Use of sentinel snails for the detection of *Schistosoma haematobium* transmission on Zanzibar and observations on transmission patterns. Acta Trop. 2013;128:234–40. 10.1016/j.actatropica.2013.01.003 23318933

[29] Frandsen F, Christensen NO. An introductory guide to the identification of cercariae from African freshwater snails with special reference to cercariae of trematode species of medical and veterinary importance. Acta Trop. 1984;41:181–202. 6206702

[30] Webster BL, Rabone M, Pennance T, Emery AM, Allan F, Gouvras A, et al. Development of novel multiplex microsatellite polymerase chain reactions to enable high-throughput population genetic studies of *Schistosoma haematobium.* Parasit Vectors. 2015;8:432. 10.1186/s13071-015-1044-6 26329827 PMC4557312

[31] Zarowiecki MZ, Huyse T, Littlewood DTJ. Making the most of mitochondrial genomes—markers for phylogeny, molecular ecology and barcodes in *Schistosoma* (Platyhelminthes: Digenea). Int J Parasitol. 2007;37:1401–18. 10.1016/j.ijpara.2007.04.014 17570370

[32] Katoh K, Rozewicki J, Yamada KD. MAFFT online service: multiple sequence alignment, interactive sequence choice and visualization. Brief Bioinform. 2019;20:1160–6. 10.1093/bib/bbx108 28968734 PMC6781576

[33] Ranwez V, Harispe S, Delsuc F, Douzery EJ. MACSE: Multiple Alignment of Coding SEquences accounting for frameshifts and stop codons. PLoS One. 2011;6:e22594. 10.1371/journal.pone.0022594 21949676 PMC3174933

[34] Crellen T, Walker M, Lamberton PHL, Kabatereine NB, Tukahebwa EM, Cotton JA, et al. Reduced efficacy of praziquantel against *Schistosoma mansoni* is associated with multiple rounds of mass drug administration. Clin Infect Dis. 2016;63:1151–9. 27470241 10.1093/cid/ciw506 PMC5064161

[35] Protasio AV, Tsai IJ, Babbage A, Nichol S, Hunt M, Aslett MA, et al. A systematically improved high quality genome and transcriptome of the human blood fluke *Schistosoma mansoni.* PLoS Negl Trop Dis. 2012;6:e1455. 10.1371/journal.pntd.0001455 22253936 PMC3254664

[36] McKenna A, Hanna M, Banks E, Sivachenko A, Cibulskis K, Kernytsky A, et al. The Genome Analysis Toolkit: a MapReduce framework for analyzing next-generation DNA sequencing data. Genome Res. 2010;20:1297–303. 10.1101/gr.107524.110 20644199 PMC2928508

[37] Stamatakis A. RAxML version 8: a tool for phylogenetic analysis and post-analysis of large phylogenies. Bioinformatics. 2014;30:1312–3. 10.1093/bioinformatics/btu033 24451623 PMC3998144

[38] Ronquist F, Teslenko M, van der Mark P, Ayres DL, Darling A, Höhna S, et al. MrBayes 3.2: efficient Bayesian phylogenetic inference and model choice across a large model space. Syst Biol. 2012;61:539–42. 10.1093/sysbio/sys029 22357727 PMC3329765

[39] Bouckaert R, Vaughan TG, Barido-Sottani J, Duchêne S, Fourment M, Gavryushkina A, et al. BEAST 2.5: An advanced software platform for Bayesian evolutionary analysis. PLOS Comput Biol. 2019;15:e1006650. 10.1371/journal.pcbi.1006650 30958812 PMC6472827

[40] Parker J, Rambaut A, Pybus OG. Correlating viral phenotypes with phylogeny: accounting for phylogenetic uncertainty. Infect Genet Evol. 2008;8:239–46. 10.1016/j.meegid.2007.08.001 17921073

[41] Sikes RS; Animal Care and Use Committee of the American Society of Mammalogists. 2016 Guidelines of the American Society of Mammalogists for the use of wild mammals in research and education. J Mammal. 2016;97:663–88. 10.1093/jmammal/gyw078 29692469 PMC5909806

[42] Morgan JA, Dejong RJ, Adeoye GO, Ansa ED, Barbosa CS, Brémond P, et al. Origin and diversification of the human parasite *Schistosoma mansoni.* Mol Ecol. 2005;14:3889–902. 10.1111/j.1365-294X.2005.02709.x 16202103

[43] Campbell G, Noble LR, Rollinson D, Southgate VR, Webster JP, Jones CS. Low genetic diversity in a snail intermediate host (*Biomphalaria pfeifferi* Krass, 1848) and schistosomiasis transmission in the Senegal River Basin. Mol Ecol. 2010;19:241–56. 10.1111/j.1365-294X.2009.04463.x 20025653

[44] Théron A. Chronobiology of trematode cercarial emergence: from data recovery to epidemiological, ecological and evolutionary implications. Adv Parasitol. 2015;88:123–64. 10.1016/bs.apar.2015.02.003 25911367

[45] Sène M, Duplantier JM, Marchand B, Hervé JP. Susceptibility of rodents to infection with *Schistosoma mansoni* in Richard-Toll (Senegal). Parasite. 1996;3:321–6. 10.1051/parasite/1996034321 9033908

[46] Catalano S, Symeou A, Marsh KJ, Borlase A, Léger E, Fall CB, et al. Mini-FLOTAC as an alternative, non-invasive diagnostic tool for *Schistosoma mansoni* and other trematode infections in wildlife reservoirs. Parasit Vectors. 2019;12:439. 10.1186/s13071-019-3613-6 31522684 PMC6745783

[47] Hipsley CA, Müller J. Beyond fossil calibrations: realities of molecular clock practices in evolutionary biology. Front Genet. 2014;5:138. 10.3389/fgene.2014.00138 24904638 PMC4033271

[48] Oleaga A, Rey O, Polack B, Grech-Angelini S, Quilichini Y, Pérez-Sánchez R, et al. Epidemiological surveillance of schistosomiasis outbreak in Corsica (France): Are animal reservoir hosts implicated in local transmission? PLoS Negl Trop Dis. 2019;13:e0007543. 10.1371/journal.pntd.0007543 31233502 PMC6611637

[49] Suzán G, García-Peña GE, Castro-Arellano I, Rico O, Rubio AV, Tolsá MJ, et al. Metacommunity and phylogenetic structure determine wildlife and zoonotic infectious disease patterns in time and space. Ecol Evol. 2015;5:865–73. 10.1002/ece3.1404 25750713 PMC4338969

