# Supplementary material for: Multihost Transmission of Schistosoma mansoni in Senegal, 2015–2018
Source: Emerg Infect Dis. 2020 Jun;26(6):1234–42. doi: 10.3201/eid2606.200107 (PMC7258455; doi:10.3201/eid2606.200107)
Supplement: Appendix — More information on multihost transmission of Schistosoma mansoni in Senegal, 2015–2018. [file 20-0107-Techapp-s1.pdf]

Article DOI: https://doi.org/10.3201/eid2606.200107

Multihost Transmission of *Schistosoma mansoni* in Senegal, 2015–2018

Appendix

Materials and Methods

Laboratory Analysis

At postmortem examination, the classification of rodents as juveniles or adults was based on body weight and reproductive status: Hubert's multimammate mice (*Mastomys huberti*) and Nile grass rats (*Arvicanthis niloticus*) weighting ≥33 g and ≥70 g, respectively, and with developed sexual traits were classified as adults (*1*). *Crocidura* shrews were identified to the genus level given the presence of multiple, morphologically undistinguishable, sympatric species in the region, while age class was not determined.

Molecular Analysis

Oligonucleotide primers for PCR and sequencing were designed by collating data for *Schistosoma* spp. available in GenBank and assembling them using CodonCode Aligner v8.0.1 (CodonCode Corporation, Centerville, MA, USA). Potential target sequences and PCR conditions were identified using primer design tools by Eurofins Genomics (https://www.eurofinsgenomics.eu/en/dna-rna-oligonucleotides/oligo-tools/primer-design-tools/). Oligonucleotide primers were supplied by Sigma-Aldrich (Sigma-Aldrich Company Ltd, Gillingham, UK). The primer pair targeting the mitochondrial 12S ribosomal RNA gene was a modified version of the primers RK12SF and RK12SR2 (*2*); it was used not only for *Schistosoma mansoni* but also for *Schistosoma haematobium* and *Schistosoma bovis* (our unpublished data). In contrast, the primer pair targeting the cytochrome *c* oxidase subunit 3 of the mitochondrial DNA (mtDNA) was specific for *S. mansoni* (Appendix Table 1). PCR cycling parameters are detailed in Appendix Table 2.

Phylogenetic Approach

12S and mtDNA sequences were concatenated, and the datasets partitioned, in SequenceMatrix v1.8 (*3*) after implementing an incongruence length difference (ILD) test (*4*) in PAUP* v4.0a164 (Sinauer Associates, Sunderland, MA, USA) to assess homogeneity between partitions. The ILD test was performed by using 1,000 replicates, random addition of sequences (10 replicates) and tree-bisection-reconnection algorithm for branch swapping. Furthermore, we tested the concatenated protein-coding mtDNA data for nucleotide saturation at each codon position in DAMBE v7.2.16 (*5,6*).

Phylogenetic analyses using maximum likelihood in RAxML v8.2 (*7*) and Bayesian inference in MrBayes v3.2.6 (*8*) invoked the substitution model indicated by the software PartitionFinder v2.1.1 (*9*) as the best-fit across the 4 partitions (noncoding and coding first, second and third codon positions). Bayesian inference in BEAST v2.5.1 (*10*) followed an initial model comparison in order to select the best-fit substitution model, clock model and tree prior. The package bModelTest v1.1.2 (*11*) further supported the selection of the HKY substitution model. Nested sampling, via the package NS v1.0.4 (*12*), was used to estimate marginal likelihood and standard deviation of each model: the strict clock (-7516.74±2.42) was selected over relaxed log-normal (-7551.55±2.57) and random local (-7557.36±2.55) clock models. All phylogenetic analyses were implemented using the Cyberinfrastructure for Phylogenetic Research web portal (https://www.phylo.org/). The resulting tree topologies were visualized using FigTree v1.4.3 (http://tree.bio.ed.ac.uk/software/figtree/).

Results

Capture Rates and Postmortem Examination

Study sites in the area of Richard Toll and the nearby lake Lac de Guiers in northern Senegal are illustrated in Appendix Figure 1. Capture rates for the small mammals trapped as part of the current study are displayed in Appendix Figure 2. At postmortem examination, 16 out of 195 (8.2%) *M. huberti* were parasitized by *S. mansoni*. The number of adult worms counted in the mesenteric vessels (present in 81.2% of the infected mice) ranged from 1 to 14 pairs (median intensity of 2 pairs), while in the portal system (present in 68.7% of the infected mice) it ranged

from 1 single male to 21 pairs and 4 single males (median intensity of 2 pairs) (Appendix Table 3).

Statistical Analysis

Differences between *Schistosoma* infection prevalence and *M. huberti* sex and age classes were tested using Pearson’s chi-squared (χ^2) test, significant when $p \leq 0.05$, in EpiTools (http://epitools.ausvet.io). Non-significant associations were found between *S. mansoni* prevalence and host traits ($p = 0.09$, $\chi^2 = 2.91$, d.f. = 1 for age; $p = 0.68$, $\chi^2 = 0.17$, d.f. = 1 for sex). However, while prevalence of *S. mansoni* seems independent of host sex, the non-significant increase with age might be due to the small population size. In fact, 15 out of the 16 *M. huberti* found infected were classified as adults, which have been previously demonstrated to be significantly more infected than juveniles (*13–15*).

Phylogenetic Analysis

The ILD test validated the combination of 12S and mtDNA data since these partitions reflected the same underlying evolutionary relationships ($p = 0.90$). The saturation plots of the protein-coding mtDNA data at model-corrected genetic distances (Appendix Figure 3) revealed that first, second and third codon positions were not saturated ($p \leq 0.05$ and index of substitution saturation between 0.01 and 0.02, smaller than its critical value which ranged between 0.75 and 0.82). Maximum likelihood and Bayesian inference yielded consensus trees with identical topologies, which strongly supported different multihost *S. mansoni* lineages circulating across the Senegal River Basin, as well as the divergence between Ugandan and Senegalese *S. mansoni* (Appendix Figure 4). The phylogenetic analysis using BEAST v2.5.1 (*10*) confirmed the presence of 4 different *S. mansoni* lineages including isolates collected from both humans and rodents. Based on the strict molecular clock, mean dates of the most recent common ancestors were 1.14 million years ago for *Schistosoma rodhaini* and *S. mansoni*, 0.19 million years ago for the sampled East and West African *S. mansoni*, and 0.13-0.02 million years ago for the sampled *S. mansoni* within Senegal (Appendix Figure 5).

References

1. Granjon L, Duplantier JM. Les rongeurs de l'Afrique sahélo-soudanienne. Marseille (France): IRD Éditions; 2009.

2. Kane RA, Southgate VR, Rollinson D, Littlewood DTJ, Lockyer AE, Pagès JR, et al. A phylogeny based on three mitochondrial genes supports the division of *Schistosoma intercalatum* into two separate species. Parasitology. 2003;127:131–7. PubMed http://dx.doi.org/10.1017/S0031182003003421

3. Vaidya G, Lohman DJ, Meier R. SequenceMatrix: concatenation software for the fast assembly of multi-gene datasets with character set and codon information. Cladistics. 2011;27:171–80. http://dx.doi.org/10.1111/j.1096-0031.2010.00329.x

4. Farris JS, Kallersjo M, Kluge AG, Bult C. Constructing a significance test for incongruence. Syst Biol. 1995;44:570–2. http://dx.doi.org/10.2307/2413663

5. Xia X, Xie Z, Salemi M, Chen L, Wang Y. An index of substitution saturation and its application. Mol Phylogenet Evol. 2003;26:1–7. PubMed http://dx.doi.org/10.1016/S1055-7903(02)00326-3

6. Xia X. DAMBE7: new and improved tools for data analysis in molecular biology and evolution. Mol Biol Evol. 2018;35:1550–2. PubMed http://dx.doi.org/10.1093/molbev/msy073

7. Stamatakis A. RAxML version 8: a tool for phylogenetic analysis and post-analysis of large phylogenies. Bioinformatics. 2014;30:1312–3. PubMed http://dx.doi.org/10.1093/bioinformatics/btu033

8. Ronquist F, Teslenko M, van der Mark P, Ayres DL, Darling A, Höhna S, et al. MrBayes 3.2: efficient Bayesian phylogenetic inference and model choice across a large model space. Syst Biol. 2012;61:539–42. PubMed http://dx.doi.org/10.1093/sysbio/sys029

9. Lanfear R, Frandsen PB, Wright AM, Senfeld T, Calcott B. PartitionFinder 2: new methods for selecting partitioned models of evolution for molecular and morphological phylogenetic analyses. Mol Biol Evol. 2017;34:772–3. PubMed http://dx.doi.org/10.1093/molbev/msw260

10. Bouckaert R, Vaughan TG, Barido-Sottani J, Duchêne S, Fourment M, Gavryushkina A, et al. BEAST 2.5: an advanced software platform for Bayesian evolutionary analysis. PLOS Comput Biol. 2019;15:e1006650. PubMed http://dx.doi.org/10.1371/journal.pcbi.1006650

11. Bouckaert RR, Drummond AJ. bModelTest: Bayesian phylogenetic site model averaging and model comparison. BMC Evol Biol. 2017;17:42. PubMed http://dx.doi.org/10.1186/s12862-017-0890-6

12. Russel PM, Brewer BJ, Klaere S, Bouckaert RR. Model selection and parameter inference in phylogenetics using nested sampling. Syst Biol. 2019;68:219–33. PubMed http://dx.doi.org/10.1093/sysbio/syy050

13. Combes C, Delattre P. Principaux paramètres de l'infestation des rats (*Rattus rattus* et *Rattus norvegicus*) par *Schistosoma mansoni* dans un foyer de schistosomose intestinale de la région Caraïbe. Acta Oecol Appl. 1981;2:63–79.

14. Duplantier JM, Sène M. Rodents as reservoir hosts in the transmission of *Schistosoma mansoni* in Richard-Toll, Senegal, West Africa. J Helminthol. 2000;74:129–35. PubMed http://dx.doi.org/10.1017/S0022149X00000172

15. Catalano S, Sène M, Diouf ND, Fall CB, Borlase A, Léger E, et al. Rodents as natural hosts of zoonotic *Schistosoma* species and hybrids: an epidemiological and evolutionary perspective from West Africa. J Infect Dis. 2018;218:429–33. PubMed http://dx.doi.org/10.1093/infdis/jiy029

16. Kane RA, Rollinson D. Repetitive sequences in the ribosomal DNA internal transcribed spacer of *Schistosoma haematobium*, *Schistosoma intercalatum*, and *Schistosoma mattheei*. Mol Biochem Parasitol. 1994;63:153–6. PubMed http://dx.doi.org/10.1016/0166-6851(94)90018-3

17. Lockyer AE, Olson PD, Østergaard P, Rollinson D, Johnston DA, Attwood SW, et al. The phylogeny of the *Schistosomatidae* based on three genes with emphasis on the interrelationships of *Schistosoma* Weinland, 1858. Parasitology. 2003;126:203–24. PubMed http://dx.doi.org/10.1017/S0031182002002792

18. Webster BL, Littlewood DTJ. Mitochondrial gene order change in *Schistosoma* (Platyhelminthes: Digenea: *Schistosomatidae*). Int J Parasitol. 2012;42:313–21. PubMed http://dx.doi.org/10.1016/j.ijpara.2012.02.001

Appendix Table 1. List of primers for PCR and/or sequencing (seq). The size of the obtained sequences is reported as number of base pairs (bp)

DNA[a]	Primer name/direction[b]	Sequence (direction 5' to 3')	bp	Use	Reference
ITS	ETTS1/F	TGCTTAAGTTCAGCGGGT	≈940	PCR/seq	(*16*)
	ETTS2/R	AACAAGGTTTCCGTAGGTGAA		PCR/seq	
12S	CL3.2/F	GTATGACTWTWGGTATTTTGC	760	PCR/seq	This study
	CL3.0/R	CARTCTAATTCTAGCGCCTG		PCR/seq	
cox1	Cox1_Schist_5'/F	TCTTTRGATCATAAGCG	≈1010	PCR	(*17*)
	Cox1_Schist_3'/R	TAATGCATMGGAAAAAAACA		PCR/seq	
cox3	CL2.1/F	AATTTGGYGGGATGAATAGC	≈1150	PCR/seq	This study
	CL2.2/R	GGAAGATCCACCAATTTACC		PCR/seq	
nad4-nad3	Sch_ND4F/F	AGNGGDTRYRTWATGAAGYTRGG	≈830	PCR	(*18*)
	Sch_ND1R/R	CCAACCTTWTTHGGNCCCTT		PCR	
	CL1.1/F	CTGAAGTTGATTCTAAGCGTTG		seq	This study

[a] Internal transcribed spacers (ITS) of the nuclear ribosomal DNA, mitochondrial 12S ribosomal RNA gene, cytochrome *c* oxidase subunit 1 (*cox1*) and subunit 3 (*cox3*), and NADH dehydrogenase subunit 4 (*nad4*) and subunit 3 (*nad3*) of the mitochondrial DNA.
[b] Directions abbreviated as F (forward) and R (reverse).

Appendix Table 2. List of cycling parameters for PCR

	PCR steps				
		34 cycles			
DNA[a]	Initial denaturation	Denaturation	Annealing	Extension	Final extension
ITS	95°C x 5 min	95°C x 30 s	56°C x 1 min	72°C x 1 min	72°C x 7 min
12S	94°C x 3 min	94°C x 30 s	50°C x 30 s	72°C x 1 min	72°C x 10 min
cox1	94°C x 5 min	94°C x 30 s	52°C x 1 min	72°C x 1 min	72°C x 7 min
cox3	94°C x 3 min	94°C x 30 s	54°C x 30 s	72°C x 2 min	72°C x 10 min
nad4-nad3	94°C x 3 min	94°C x 30 s	56°C x 30 s	68°C x 2 min	68°C x 10 min

[a] Internal transcribed spacers (ITS) of the nuclear ribosomal DNA, mitochondrial 12S ribosomal RNA gene, cytochrome *c* oxidase subunit 1 (*cox1*) and subunit 3 (*cox3*), and NADH dehydrogenase subunit 4 (*nad4*) and subunit 3 (*nad3*) of the mitochondrial DNA.

Appendix Table 3. Universal Transverse Mercator coordinates of each sampling site and proportion of hosts infected with *Schistosoma mansoni* (median and range intensity estimates of individual counts of adult parasites are reported in parentheses)

		Mastomys huberti		*Arvicanthis niloticus*	*Crocidura* sp.
Sampling site	Coordinates	Juveniles (n = 46)	Adults (n = 149)	Adults (n = 42)	(n = 14)
Diaminar	28Q 403440 1770923	0/1	0/4	—	—
Diokhor	28Q 405650 1790070	0/3	0/13	0/2	—
Feto	28P 396044 1762392	0/5	0/5	0/1	—
Foss	28Q 409514 1786812	—	0/7	0/5	0/4
Ganket	28P 400370 1767221	—	2/4 (18.5; 5-32)	0/4	—
Gueo	28P 398984 1762405	1/4 (2)	9/15 (17; 3-64)	—	—
Guidick	28Q 403218 1781600	—	—	—	—
Keur Momar Sarr	28P 396747 1761044	0/7	1/12 (2)	—	—
Malla	28Q 401755 1785376	—	0/4	0/2	—
Mbane	28Q 414315 1799080	0/2	0/10	0/1	0/1
Mbrar	28Q 400616 1773916	0/5	0/5	0/2	—
Merina Guewel	28P 395943 1762998	0/3	1/9 (2)	—	—
Nder	28Q 406603 1798615	0/3	0/5	0/6	—
Ndiakhaye	28Q 411879 1790310	—	0/5	0/1	0/1
Ndieumeul	28Q 408200 1793208	0/4	0/16	0/3	—
Ngnith	28Q 402619 1788643	0/2	0/13	0/10	—
Pomo	28Q 413482 1793292	0/1	0/4	—	0/3
Saneinte	28Q 414316 1795777	—	0/1	0/2	—
Syere	28Q 404176 1777690	—	0/1	—	—
Temeye	28Q 417538 1806038	0/6	2/16 (3; 2-4)	0/3	0/5
Yamane	28Q 394854 1780516	—	—	—	—

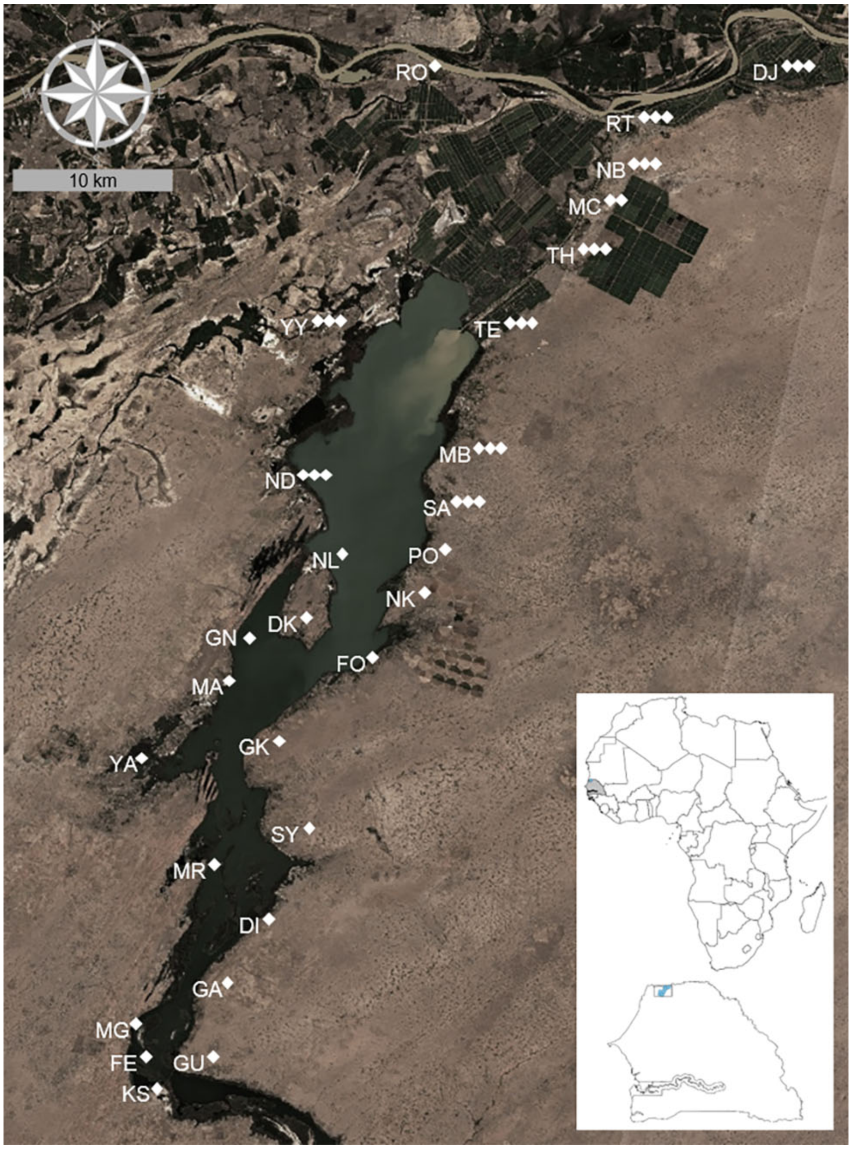

Appendix Figure 1. Map of study sites in northern Senegal. Three white diamonds indicate sites where parasitological surveys of humans, small mammals and snails were conducted. Two white diamonds indicate localities where only humans and snails were sampled. A single white diamond shows sampling sites where only small mammals were surveyed. Map includes trapping sites of small mammals investigated during a previous survey (*15*). DI, Diaminar; DJ, Didjiery; DK, Diokhor; FE, Feto; FO, Foss; GA, Ganket; GK, Guidick; GN, Ngnith; GU, Gueo; KS, Keur Momar Sarr; MA, Malla; MB, Mbane; MC, Medina Cheikhou; MG, Merina Guewel; MR, Mbrar; NB, Ndombo; ND, Nder; NK, Ndiakhaye; NL, Ndieumeul; PO, Pomo; RO, Rosso; RT, Richard Toll; SA, Saneinte; SY, Syere; TE, Temeye; TH, Thiago; YA, Yamane; YY, Yetti Yone.

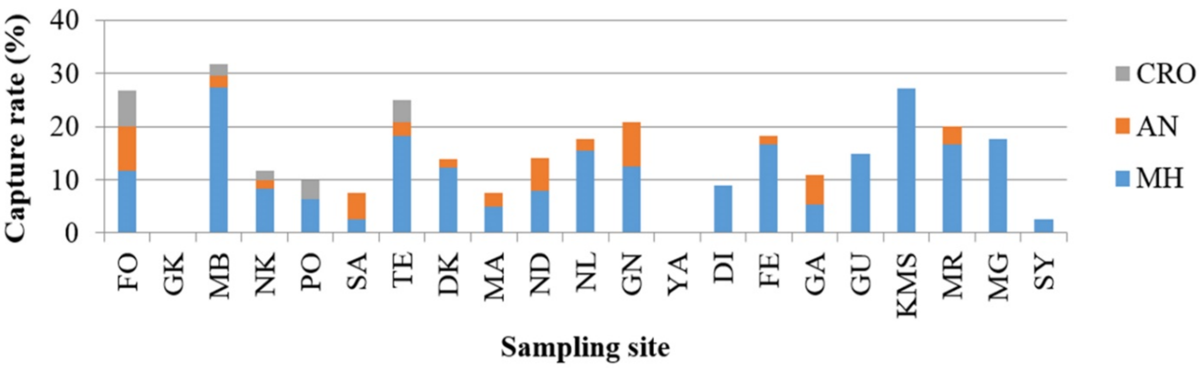

Appendix Figure 2. Capture rates (calculated as the proportion between captures and active traps set overnight) for *Mastomys huberti* (MH), *Arvicanthis niloticus* (AN) and *Crocidura* sp. (CRO) at each sampling site situated on the shores of the lake Lac de Guiers, Senegal. DI, Diaminar; DK, Diokhor; FE, Feto; FO, Foss; GA, Ganket; GK, Guidick; GN, Ngnith; GU, Gueo; KMS, Keur Momar Sarr; MA, Malla; MB, Mbane; MG, Merina Guewel; MR, Mbrar; ND, Nder; NK, Ndiakhaye; NL, Ndieumeul; PO, Pomo; SA, Saneinte; SY, Syere; TE, Temeye; YA, Yamane.

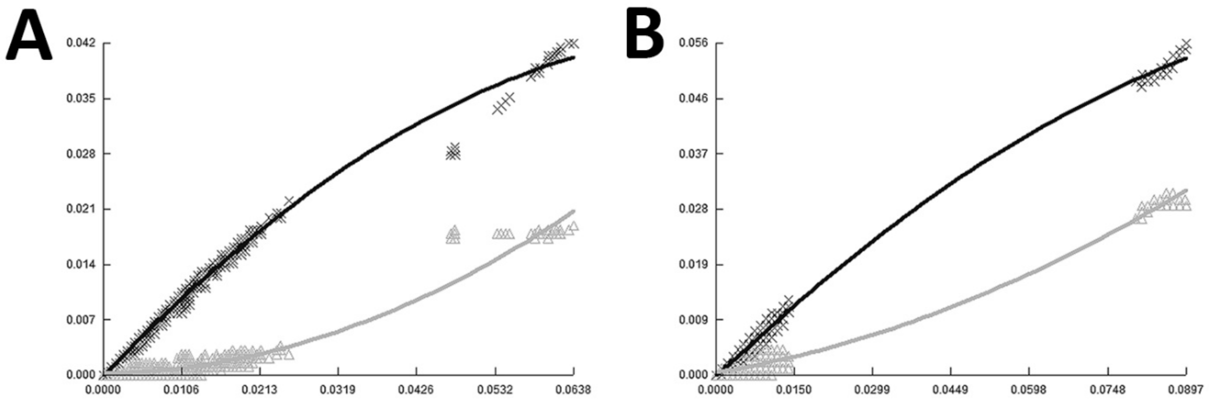

Appendix Figure 3. Saturation plots for first and second codon positions (A), and third codon position only (B), of the concatenated protein-coding sequences of the mitochondrial DNA (2,874 base pairs). Transitions (black crosses and curve) and transversions (grey triangles and curve) are plotted along the y-axis against K80 genetic distance (x-axis).

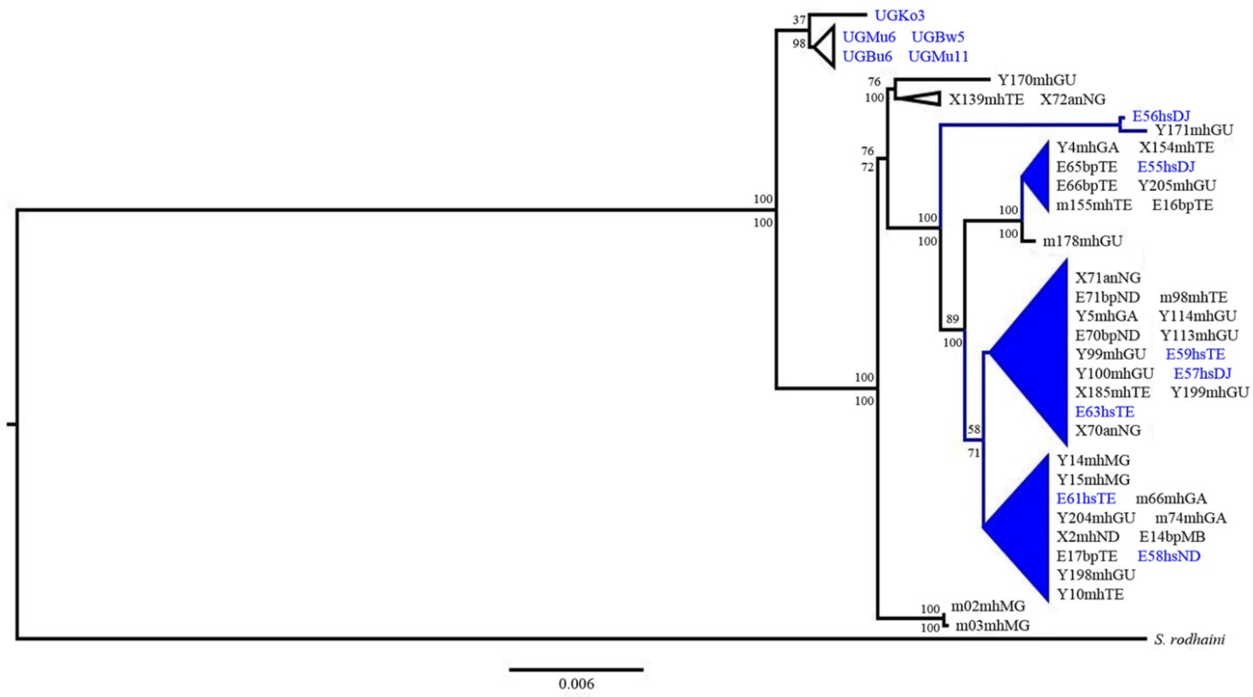

Appendix Figure 4. Maximum likelihood (ML) and Bayesian inference (BI) of relationships among *Schistosoma mansoni* isolates, with *Schistosoma rodhaini* used as outgroup, based on the concatenated mitochondrial 12S ribosomal RNA and DNA (*cox1*, *cox3*, *nad4*, *nad3*) sequences. Support from bootstrap replicates (ML) and posterior probabilities (BI) is indicated above and below each node, respectively. Branches were collapsed with nodal support ≤70% for both ML and BI analyses. The blue color highlights *S. mansoni* from humans and clades including isolates from both humans and rodents (either *Mastomys huberti* or *Arvicanthis niloticus*). Scale bar indicates number of nucleotide substitutions per site.

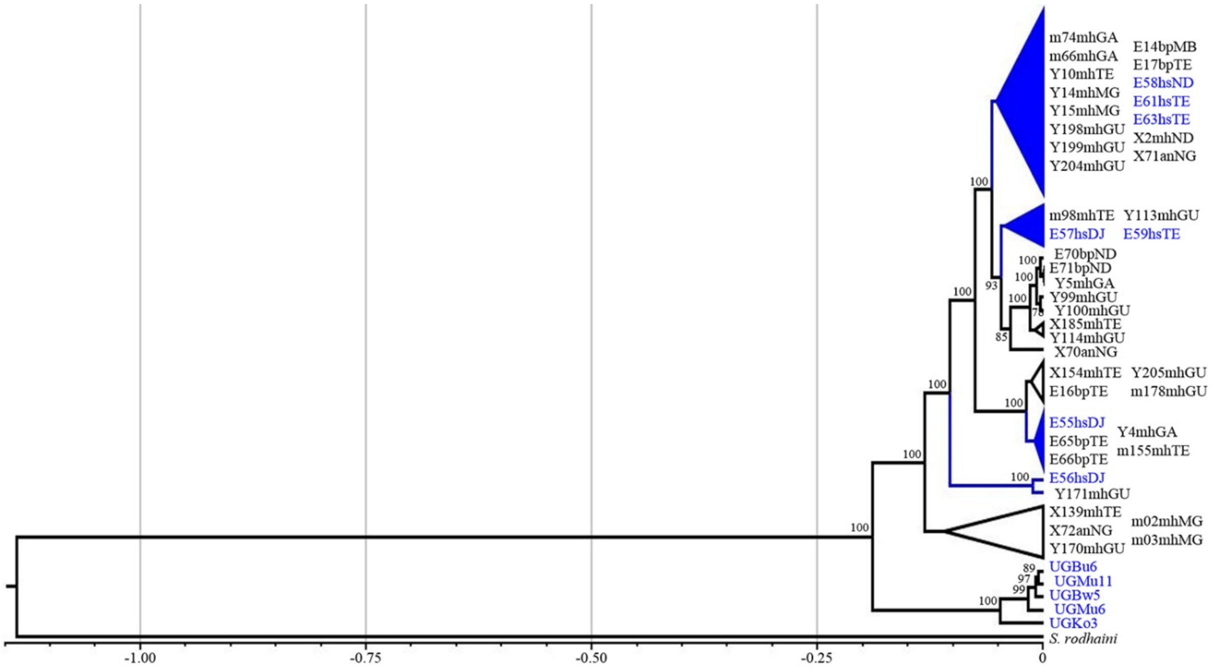

Appendix Figure 5. Bayesian phylogenetic tree of *Schistosoma rodhaini* and *Schistosoma mansoni* isolates based on the concatenated mitochondrial 12S ribosomal RNA and DNA (*cox1*, *cox3*, *nad4*, *nad3*) sequences. Support from posterior probabilities is indicated for each node (branches were collapsed with nodal support ≤70%). The blue color highlights *S. mansoni* from humans and clades including isolates from both humans and rodents (either *Mastomys huberti* or *Arvicanthis niloticus*). The timescale is in million years ago.
